# Videoconferencing-Based Telemental Health: Important Questions for the COVID-19 Era From Clinical and Patient-Centered Perspectives

**DOI:** 10.2196/24021

**Published:** 2020-12-08

**Authors:** Emil Chiauzzi, Ashley Clayton, Jina Huh-Yoo

**Affiliations:** 1 Tridiuum Inc Philadelphia, PA United States; 2 Yale School of Medicine Yale University New Haven, CT United States; 3 Department of Information Science College of Computing and Informatics Drexel University Philadelphia, PA United States

**Keywords:** telehealth, telemental health, COVID-19, videoconferencing, ethics, privacy, mental health, psychotherapy, patient-centered, lived experience

## Abstract

The COVID-19 pandemic has intensified the search for digital approaches in mental health treatment, particularly due to patients and clinicians practicing social distancing. This has resulted in the dramatic growth of videoconferencing-based telemental health (V-TMH) services. It is critical for behavioral health providers and those in the mental health field to understand the implications of V-TMH expansion on the stakeholders who use such services, such as patients and clinicians, to provide the service that addresses both patient and clinical needs. Several key questions arise as a result, such as the following: (1) in what ways does V-TMH affect the practice of psychotherapy (ie, clinical needs), (2) to what extent are ethical and patient-centered concerns warranted in terms of V-TMH services (ie, patient needs), and (3) how do factors related to user experience affect treatment dynamics for both the patient and therapist (ie, patient and clinical needs)? We discuss how behavioral health providers can consider the future delivery of mental health care services based on these questions, which pose strong implications for technological innovation, the adaptation of treatments to new technologies, and training professionals in the delivery of V-TMH services and other digital health interventions.

## Introduction

Videoconferencing-based telemental health (V-TMH) has been the subject of clinical and research discussions for many years. Since the advent of the COVID-19 pandemic however, it has entered public health discussions in a significant way [1,2]. Essentially, V-TMH services operate on platforms used for business meetings (eg, Doxy.me, Vidyo, VSee, and Thera-link), allowing users to connect to each other via their desktop computers or mobile devices. A number of these platforms are integrated into electronic health records (EHRs), which allow for the use of existing workflows, record-keeping systems, and portals [3]. To facilitate treatment, these platforms often include scheduling and check-in systems and so-called “waiting rooms” so that clients can begin their sessions when the clinician is available, as well as high-definition audio and video, adherence to Health Insurance Portability and Accountability Act (HIPAA) data privacy requirements, branding, and analytics. Some platforms do not require the user to download software or plugins, but simply use a secure browser-based link. For HIPAA-compliant platforms, audio and video communications are encrypted, no recordings are stored, and no personal health information is collected unless an EHR interface is used.

Despite clinician concerns about adverse effects on the therapeutic alliance, studies have indicated high ratings of satisfaction and therapeutic alliance from both patients and clinicians [4,5]. Several systematic reviews have supported the efficacy of V-TMH and telehealth in general, indicating comparable effectiveness with face-to-face treatment for a variety of conditions (eg, anxiety, depression, posttraumatic stress disorder, and substance abuse) and age groups (eg, adult, child, and geriatric) [6,7]. However, although there is empirical support for delivering cognitive behavioral therapy through telehealth services, there is still limited information about which therapy modalities are most amenable to telemental health care delivery [7]. Additional data are needed to assess clinicians’ and patients’ satisfaction with more relationally focused interventions, such as psychodynamic and interpersonal therapy [8].

Until recently, the use of telemental health in psychiatry was progressing at a relatively slow pace. Practicing psychologists had only engaged in the sporadic use of telecommunication technologies, such as videoconferencing (25%), instant messaging (3%), or smartphone apps (7%), and had spent less than 10% of their time delivering web-based psychotherapy [9]. Furthermore, only 5% of psychiatrists treating Medicare patients have conducted at least 1 telemedicine visit [10]. Many care providers lack technical knowledge, are not incentivized financially to provide telemental health services, or are concerned about safety and security issues with such services [9,11,12].

Before the COVID-19 pandemic era, care providers and institutions generally implemented V-TMH by choice. However, physical distancing regulations require most nonacute psychiatric care to be delivered remotely, resulting in the dramatic growth of telemental health services. For example, the Blue Cross Blue Shield of Massachusetts Foundation reported that the number of daily telehealth claims increased from 200 in February 2020 to 38,000 in May 2020, and about half of all telehealth claims were for behavioral health services [13].

These trends have increased the urgency to examine the effects of telemental health expansion on those who directly use telemental health services—patients and clinicians. The focus in current literature has been on demonstrating the equivalence of outcomes between in-person and telemental health care. Although these outcomes may be similar, it has become increasingly clear that the experience is different for all concerned. Reports published since the beginning of the COVID-19 pandemic have suggested that V-TMH alters the treatment dynamics in psychotherapy, introduces new ethical concerns, and shapes new interactions between clinicians, patients, and technology. Accordingly, we explored 3 perspectives that are particularly involved with V-TMH—clinical, ethical, and human-computer interaction perspectives. This viewpoint article addresses the following 3 questions: (1) in what ways does telemental health affect the practice of psychotherapy, (2) to what extent are ethical and patient-centered concerns warranted in terms of video-based therapy services, and (3) how do human-computer interaction factors affect treatment dynamics for both the patient and therapist? This paper should not be read as a literature review, but as an opinion piece that addresses the important treatment implications of the expanded use of V-TMH resulting from the COVID-19 pandemic. Our objective is to acknowledge and address the experiences of both clinicians and patients in order to optimize this very promising technology.

## V-TMH and the Practice of Psychotherapy

### Advantages of V-TMH

Previous studies on V-TMH have noted the convenience of this technology. As V-TMH has improved, people in rural and underserved areas have been able to access care more readily [14]. V-TMH helps overcome mobility issues due to age or disabilities, scheduling problems, and limitations in transportation [15]. Furthermore, people who are concerned about the stigma of being seen at a mental health clinic or those who want greater privacy than what face-to-face services can offer may prefer V-TMH [15]. V-TMH may also be particularly advantageous to women due to their child care, spousal care, and older adult caregiving responsibilities [16]. The effectiveness of telemental health also crosses generational lines, as it has been shown to improve behavioral health access for children and adolescents [17] and has been well-accepted in geriatric inpatient and nursing home consultations [18]. Additionally, recent accounts have indicated that V-TMH enables clinicians to maintain clinical volumes despite social distancing mandates, allows patients to attend sessions when they cannot leave home due to health reasons or scheduling conflicts, and reduces no-show rates [1]. Based on these studies, it is clear that V-TMH has strong potential in retaining those who already receive mental health services and attracting new patients who might be unable to access face-to-face services.

There is also evidence that indicates V-TMH has a number of benefits beyond convenience and easy access. V-TMH is appropriate for a broad range of mental health patients, but moderators for determining which patients and patient settings may benefit the most from V-TMH services have yet to be determined [19]. At this point, it appears that that the benefits of V-TMH outweigh its deficiencies, but the former can become the latter unless clinical adaptations are considered. These adaptations apply to clinical conditions that may be more amenable to V-TMH, strategies for managing serious mental illnesses or behavioral issues, and considerations in conducting formal assessments and psychological testing.

### Diagnostic Considerations

Telemental clinical outcomes are comparable to conventional treatment outcomes across diverse patient diagnostic groups [20]. V-TMH may be particularly advantageous for patients with conditions that detract from patient involvement in office-based treatment. These conditions include severe anxiety and avoidant behavior, severe trauma-related disorders, and agoraphobia [15]. Telemental care may be preferable for patients with severe anxiety disorders [2,19]. Telemental care may also be preferable for patients whose behaviors pose a risk to others, such as those with violent/homicidal tendencies or a history of sexual assault [15]. We will address the management of safety issues further in the Ethical Considerations in V-TMH section of our discussion.

### Addressing Serious Mental Illness

Although people with serious mental illnesses have a lower rate of smartphone ownership than those without serious mental illnesses, the ability to use apps and social media is comparable between the 2 groups [21]. Despite a variety of clinical challenges, there is a consensus that V-TMH may be feasible, usable, and acceptable for people with serious mental illnesses [22-24]. Promising outcomes for self-management, medication adherence, psychoeducation, and symptom monitoring have been obtained from a broad set of telemental and mental health apps [21]. The use of V-TMH has also been shown to reduce self-reported psychiatric symptoms, emergency room visits, and hospital admissions [25]. However, despite these positive outcomes, a review on the use of telepsychiatry for a broad range of psychiatric disorders (eg, schizophrenia, schizoaffective disorder, and bipolar disorder) found little evidence that this modality affected hospital readmission rates [26]. The American Psychological Association (APA) Task Force on Serious Mental Illness/Severe Emotional Disturbance recommends several strategies, as follows: (1) practice sessions to familiarize patients with telemental health technology, (2) the use of brief and frequent sessions to manage periods of distress and reinforce the therapeutic alliance, and (3) the promotion of team collaboration to help patients maintain access to prescriptions and lab testing [27].

### Assessment and Psychological Testing

The administration of interview-based assessments and psychological testing through V-TMH presents unique challenges. Psychological testing procedures that require the physical manipulation of materials, standardized interactions, or observations in a physical environment may require adaptations [28]. This is particularly true for cognitive or neuropsychological assessments, which may be sensitive to the lack of a physical presence and the technological comfort of the tester and patient [29]. Many of these assessments may be time sensitive or involve high stakes. Therefore, there is a need to identify potential factors that influence test reliability and validity and find ways to account for them so that testing procedures can be optimized [29].

Emerging findings under controlled conditions have suggested that equivalence between face-to-face and videoconference-based assessments can be attained [30]. This has been demonstrated with brief cognitive test batteries [30], and obtaining such equivalence may work better with neuropsychological testing involving visually-dependent tasks rather than motor-dependent tasks [31]. When providing V-TMH services, the APA suggests carefully considering how V-TMH affects the presentation of materials (eg, shadowing and blurriness), substituting subtests that do not require the physical manipulation of objects, and widening confidence intervals when interpreting results [28]. In recent years there has been a proliferation of mobile technology–based cognitive assessments that have demonstrated psychometric properties comparable to those of laboratory-based assessments [32]. These apps can support repeated measurements that increase detection sensitivity in a patient’s natural environment and can supplement more formal traditional methods.

## Ethical Considerations in V-TMH

The forced use of V-TMH during, and potentially after, the COVID-19 global pandemic raises ethical concerns about patient safety and privacy.

### Patient Safety Risk

One of the primary ethical considerations in V-TMH is adequately ensuring the safety of the patient. Suicide prediction is challenging even under ideal assessment conditions, and it may be more difficult through V-TMH services. Psychiatrists and the mental health community have invested in the development of tools and predictive models to help determine who is at an elevated risk of suicide, but little success has been achieved [33-35]. Relying on clinical intuition to assess suicidality is less than ideal in face-to-face encounters, but V-TMH may introduce subtle changes to interpersonal relationships, trust, and decision making. For example, distortions or delays in the video can increase anxiety and feelings of disconnection in patients experiencing crises [36].

Due to the difficulty in determining treatment allocation based on suicide assessments [34], high-stakes decisions, such as seeking the involuntary detainment of a patient, require careful consideration. Involuntary commitment may be a tragic necessity during a psychiatric emergency, and it can cause potential harm and suffering for patients [37]. One of the most difficult decisions—if not the most stressful decision—for any physician or licensed mental health provider can make is to infringe on an individual’s fundamental civil rights; however, there is precedence. In 1993, a patient was involuntarily committed to a psychiatric hospital by use of videoconferencing. The patient petitioned that his rights to due process were violated, alleging that “the quality of information available through videoconferencing was limited and increased the risk of erroneous result” [38,39]. Upon appeal, the court determined that videoconferencing was equal to a face-to-face evaluation, asserting that the patient’s facial expressions and demeanor were easily observable. Therefore, videoconferencing did not increase the risk of erroneous results [39]. 

We do not yet know how V-TMH impacts clinicians’ ability to predict who is acutely at risk of suicide. However, actions can be taken to assist in managing such risks. Guidelines produced by the APA and American Telemedicine Association provide best practices for managing suicide risk [2]. Many of the guidelines mirror those for in-person care, such as obtaining patient contact information; monitoring the patient’s risk factors, use of substances, and access to lethal means; and having a safety plan available [40,41]. The major difference between V-TMH and in-person care is the use of telehealth technologies, as an interruption in a connection between the clinician and patient at an inopportune time can interfere with a needed intervention. The APA guidelines stress the need for the identification and consistency of the patient’s location during sessions and the availability of multiple forms of contact information (eg, phone, email, and SMS texts) in case contact is interrupted [2]. Despite these guidelines, clinicians remain skeptical about whether mental health professionals can screen at-risk patients through telemental health methods [9]. At present, it is incumbent on providers to carefully consider the risks involved in client safety and liberty when providing remote services to treat individuals in acute crises.

There has been a resurgence of enthusiasm for eased access to both in-home and cross-state telehealth care [42]. We must carefully consider the significant implications that such a model would have on individuals at elevated risk of suicide, especially when there is a heightened potential for detainment and involuntary hospitalization. Enlisting a second care provider who is geographically closer to the client and building relationships with local services can help clinicians provide telemental health services across state lines and increase patient safety [43].

### Intimate Partner Violence

There is limited data on the safety and efficacy of telemental health treatment for individuals who actively experience intimate partner violence (IPV) [44]. A systematic review of studies on using V-TMH for people who have experienced IPV only found a small number of studies, and most of these studies focused on women [44]. Other such studies on telehealth were designed for women in domestic violence shelters or mental health clinics [45,46]. There are no data available on providing V-TMH interventions at home for this population, and the risks and unintended negative consequences of V-TMH interventions are currently unknown.

It has been reported that people who have experienced previous natural disasters (eg, Hurricanes Harvey and Katrina), shelter-in-place orders, restrictions on travel, increased basic needs insecurity, and loss of jobs are likely to experience IPV [47,48]. Individuals who experience violence at home need access to support and services. For individuals who received mental health treatment before the COVID-19 pandemic, the transition to telemental health services is exceedingly complicated. Perpetrators of IPV often closely monitor and restrict access to electronic communication, making it difficult for individuals who experience IPV to access help, especially while at home. Such individuals who can attend V-TMH appointments may be unable to obtain any privacy at home, making it extremely difficult to disclose IPV experiences and placing them in imminent danger. Similarly, the telehealth provider must be scrupulous with their words, lest they say something that would upset the perpetrator and elevate the risk of danger. Data regarding the safety of V-TMH at home are not yet available. However, crisis support and other such lines have been supporting individuals who experience IPV for decades.

It is easier to move around and find privacy while on the phone than doing so while in a videoconference, and the care provider on the other end cannot be seen during phone calls. Therefore, covers such as “I’m talking to my sister” can be used. As such, phone calls may be safer than V-TMH. At present, there is no empirical evidence that favors video-based care over audio-only care [1]. However, voice-only communications may enhance empathic accuracy and the assessment of emotions [49]. Many insurers have temporarily allowed mental health providers to bill for telephone-delivered services during the COVID-19 pandemic, and this should be viewed as a viable option. However, as temporary policy relaxations begin to roll back while the public health crisis continues, it is essential to carefully consider how different communication modalities impact access to care and support for vulnerable populations.

### Protecting Privacy

Confidentiality is a core obligation and ethical standard for health care providers, especially for mental health clinicians who by the nature of their work treat vulnerable and historically marginalized — and perhaps presently marginalized — populations. Breaches of confidentiality within mental health care can decrease the efficacy of therapy [50]. The US government temporarily relaxed HIPAA regulations, which allowed for the quick scaling of telehealth care to increase access to medical care while practicing shelter-in-place orders and physical distancing guidelines during the COVID-19 pandemic. The loosening of regulations correctly prioritized access to care over the tools that enabled it [51].

Several V-TMH services, especially those provided by hospitals and large health systems, now use EHRs. Before the COVID-19 pandemic, there was some integration of psychiatric care into EHRs, yet confidentiality remains an issue. Documenting diagnoses and treatment plans within the EHR helps ensure that care providers are on the same page. However, the majority of EHRs do not have a section with restricted access for care providers to write confidential, detailed notes about an encounter, thereby violating federal statute 42 of the Code of Federal Regulations Part 2 [52]. This statute was intended to protect patient privacy and avoid adverse outcomes, but its unintended consequences have made integrated care difficult. While the HIPAA allows for the sharing of patient information for care coordination, the sharing of psychiatric notes falls into ethical and legal grey areas. Care providers should openly discuss the unique limitations of privacy when using an EHR, even if these limitations are described within written informed consent forms [53]. With the lightning-speed implementation of telemental health services, care providers must take the time to research and share the benefits and risks of V-TMH with their patients, especially those that differ in face-to-face treatments. This is critical when using non-HIPPA-compliant platforms during the pandemic. Since informed consent requirements vary state by state and not all states require additional consent, care providers need to stay attuned to changes in regulations.

## User Experience With V-TMH

### Clinician Perspectives

In 1 survey, more than 75% of psychologists were slightly or not at all confident that they could use telecommunication modalities (ie, video, phone, email, and chat and messaging apps) without an initial in-person assessment [9]. With the widespread uptake of telemental health during the COVID-19 pandemic, we are gaining a better understanding of everyday clinicians’ experiences with providing treatment via V-TMH instead of just their intentions or attitudes toward hypothetical V-TMH situations. Recent interviews with psychiatrists who deliver telemental care during the COVID-19 pandemic have found that clinical challenges, such as the inability to conduct physical examinations, difficulties in evaluating extrapyramidal symptoms from antipsychotics, and home distractions, may negatively impact the quality of provider-patient interactions [54]. These challenges may be partially due to the Ryan Haight Act regulations that previously required psychiatrists to conduct an initial in-person medical evaluation prior to prescribing controlled substances. However, as of March 2020, the Drug Enforcement Administration has suspended these regulations, thereby requiring psychiatrists to adjust to remote assessments when prescribing controlled medications. On the positive side, psychiatrists and other clinicians have reported increases in their understanding of family and home dynamics, access to underserved patients, and their ability to facilitate treatment when patients are more relaxed in their homes [1].

Beyond the clinical advantages and disadvantages of V-TMH, clinicians have also been sharing their individual experiences related to the phenomenological aspects of V-TMH. Recent newspaper and magazine articles have highlighted the newfound intimacy that telemental health has introduced to some therapist-client relationships. However, this newfound intimacy may not be welcomed by all. Like their patients, clinicians have had to optimize workspaces to balance professionalism and privacy, particularly when their own family members may be at home due to work and school closures [1]. In Self Magazine, Dr Jessica Gold recently shared her experiences as a psychiatrist now practicing teletherapy and she highlighted the “very significant things” she misses about in-person treatment, as follows: “It turns out I went into a field of talking to humans and listening to them because, quite simply, I like people. Online interactions aren’t the same” [55]. Furthermore, a recent article in The New Yorker features multiple psychologists discussing their new consciousness of patients seeing them and highlighting the difficulty of holding the therapeutic frame without the regular rituals of in-person care [56]. Additionally, therapists have reported the disconcerting and sometimes distracting experience of seeing their own faces during videoconferencing. A mental health clinician in Washington, DC experienced the loss of a safe space and spoke about how the transition from the refuge of the therapy office to receiving therapy within one’s home is difficult for both therapists and clients alike [57].

### Patient Perspectives

Evaluations of patients’ attitudes toward telehealth have focused primarily on satisfaction data and therapeutic alliance ratings [8]. Although important, these are only 2 factors for assessing patients’ satisfaction [8]. As V-TMH takes on an increasingly influential role in mental health treatment during the COVID-19 pandemic, it is useful to consider patient accounts that reflect the less easily measured aspects of V-TMH interactions. V-TMH brings nuanced contexts that might interfere with the quality and privacy of mental health therapy sessions.

In many cases, clinicians may not be aware of the practical decisions and dilemmas that their patients face when attending these sessions. For instance, finding a private and comfortable space at home may force individuals to consider using private, intimate spaces. Due to a lack of privacy within some homes, patients have taken to using their bathrooms and closets for telehealth visits [58]. Some have also chosen to seek privacy in their cars or out on walks [59].

The use of V-TMH may also cause patients to feel unanticipated self-consciousness and discomfort during V-TMH sessions. This may potentially affect self-disclosure or cause heightened emotional reactions. Self-disclosure is defined as “the verbal revealing of personal information, thoughts, or feelings about oneself” [60], and it is one of the most salient behaviors in computer-mediated communication (CMC) [61]. Self-disclosure plays a critical role in relationship development and maintenance in CMC and face-to-face relationships [62]. Depending on how comfortable patients are with self-disclosure during V-TMH appointments, the results may be controversial, similar to how there is disagreement in literature about the consistency, size, and direction of differences of self-disclosure in CMC [61].

Self-view is a unique V-TMH feature that allows for the viewing of one’s own face during conversations. Since this is dramatically different from in-person conversations and treatment, the self-view feature may interfere with participants’ self-disclosure during V-TMH appointments. In most cases, the visual anonymity provided by CMC has fostered significantly higher self-disclosure levels than in face-to-face conversations [63]. However, the self-view feature of V-TMH tools directly counteracts the critical role that CMC plays in self-disclosure. Furthermore, V-TMH generates an environment in which gestural interactions and nonverbal cues (eg, eye contact) may be distorted and inadequately communicated due to technological factors, such as video bandwidth, camera viewpoint, and image resolution quality [64-67]. Technological features that might be considered minor, such as screen size, can affect perceptions of trust toward objects shown in the video calls [68]. Additionally, a sense of proximity can be gained through various methods, such as matching the patient’s eye gaze or showing more of the clinician’s body in video calls as opposed to just a headshot. This sense of proximity can help provide an effective V-TMH experience [69]. As such, features in video calls, such as the background shown behind the speaker and being able to see one’s own face, can be moderating factors for establishing affective and cognitive trust, a sense of proximity, good telepresence, and social and emotional connections.

People will eventually get accustomed to the affordances available in V-TMH technologies and learn to better regulate their interactions [70]. However, during the COVID-19 era, patients lack options for seeking mental health services and may feel forced to use V-TMH services over other modalities because of public health regulations. This raises several unresolved questions and many implications to consider when thinking about the post-COVID-19 era.

## Implications of V-TMH in the Peri- and Post-COVID-19 Era

### Current Status of V-TMH

Since the COVID-19 pandemic has forced patients and clinicians to shift quickly from face-to-face mental health therapy to V-TMH therapy, the focus of V-TMH has turned to implementation and access. This has allowed little consideration for the nuances of the V-TMH treatment experience, leaving many unresolved questions that software developers, health care administrators, organizations, patients, and clinicians need to consider. As we navigate through the current pandemic and prepare for a post-pandemic recovery, these questions are highly relevant to the future viability of V-TMH.

### How Can V-TMH Services Assist Those Whose Mental Health Is Affected by COVID-19?

Past studies on severe acute respiratory syndrome survivors have found increases in the incidence of posttraumatic stress disorder, depression, chronic pain, obsessive-compulsive disorder, and chronic fatigue syndrome [71]. Negative quarantine effects increase with the perceived difficulty of compliance, length of quarantine, compliance with quarantine requirements, fear of infection, lack of supplies, and financial pressures [72]. Unemployment is also of particular concern. Past data have shown that a 5% increase in unemployment is associated with 4000 additional suicides and 775 additional deaths for each additional percentage point [73]. The Well Being Trust estimates that between 27,644 and 154,037 additional deaths have been due to suicide and substance abuse, and these additional deaths are related to the effectiveness of recovery efforts [74]. These data show the high demand for mental health services. Future models of mental health care delivery, including V-TMH, will need to address the influx of new cases by providing more flexible treatment options. Since mental health clinicians have been treating patients during the pandemic, many clinicians have gained experience in addressing both COVID-19–related and mental health–related challenges through V-TMH. Cost-effective, readily available, and socially distanced treatment will be necessary for those dealing with mental health crises, the aftereffects of quarantine, unemployment, and social adjustment issues. Since many clinicians have backgrounds in behavior change techniques, they may also have a role in supporting the behavioral strategies used to prevent the transmission of SARS-CoV-2 (eg, mask use, hand washing, etc) [75].

### How Can We Better Address the Digital Divide by Helping Patients to Effectively Use Technology?

Since most Americans, including those who previously lacked internet access, are increasingly gaining access to the internet through smartphones, V-TMH has the potential to become more readily available. Telehealth has typically been viewed as a service that is useful for hard-to-reach locales (eg, rural areas) or locations with underserved populations. This has shown no signs of abating. For example, since the pandemic began, the telehealth adoption rate for primary care visits was 28% higher in urban areas than in rural areas [76]. However, telehealth adoption rates in the Medicaid population are lower than those in populations who do not have Medicaid [76]. Due to the COVID-19 pandemic, there is a broader need for accessing telehealth care among mental health patients, as evidenced by the 61.8% telehealth adoption rate among psychiatrists [77].

Solutions for the digital divide issue must be more nuanced than providing mere access to the internet, as improved access to broadband internet is critical [78]. The quality of one’s connection (ie, high-speed vs low-speed bandwidth) brings more richness and variation to the internet experience [79]. With V-TMH, it is about making software usable and relevant to a broad audience, thereby enabling active participation in web-based discussions and the interpretation of social cues from an interpersonal context that is more limited than in face-to-face sessions.

Social determinants of health, such as cultural expectations about technology, digital literacy, economic factors, and comorbidities that affect the use of technology, may perpetuate inequalities [80]. Programs, such as the Digital Opportunities for Outcomes in Recovery Services (DOORS), have been developed to address low digital literacy in patients with serious mental illnesses [81]. This interactive training program helps patients learn safe smartphone usage, use technology to build wellness habits, and learn new skills through web-based resources. Although DOORS was specifically designed to support the use of digital health apps, its guiding principles and framework could easily be adapted to support the use of V-TMH services. Older adults who live in assisted and independent living communities and have basic training on using computers and navigating through the internet are able to reduce loneliness and increase social contact [82]. These findings provide critical evidence for how technology training can resolve negative outcomes that stem from the isolation that vulnerable populations may face with the COVID-19 quarantine. Beyond addressing the digital literacy gap, it is also important to note that the 2018 American Community Survey found that 15% of households lack broadband internet access, and a 2020 Brookings Report asserted that “broadband is the country’s most inequitable infrastructure” [83]. Expanded access can be achieved through a government subsidy program, similar to the Federal Communications Commission Lifeline program, but additional solutions are needed.

### What Have We Learned About the User Experience and Clinical Implications for Those Who Have Transitioned From Face-To-Face Treatment to V-TMH Treatment?

Treatment effectiveness depends on how well clinicians and patients can adapt to new ways of delivering and receiving mental health services. New technology adoption is not simply driven by efficiency or technological advancement, it is also governed by multiple contextual factors, including the meanings that people put on such factors. The implications of particular gestures portrayed by a limited view of a person’s face and chest differ from those portrayed by hands and legs [65]. Beyond the lack of V-TMH quality due to limited internet bandwidth, the more important problem lies in how the contexts delivered through V-TMH are being perceived and how people interpret what it means to use V-TMH for the purpose of mental health therapy. What would it mean to not meet the care provider in person anymore? What does it mean to blend the therapy hour into one’s daily schedule instead of having delineated time in a professional office? For clinicians, what does it mean to not be able to physically hand over a tissue box to the patient? How do the meanings that people put on a technological tool affect treatment outcomes? These are questions that we have not dealt with on the scale we have been anticipating. Studies on the effectiveness of mental health service adaptations during the COVID-19 pandemic remains mainly anecdotal, and quality measures for assessing effectiveness have yet to be proposed [84].

### Given the Popularity and Efficacy of V-TMH, What Are the Privacy Implications for Loosening Post-COVID-19 Federal and State Regulations Governing V-TMH Delivery and Reimbursement?

During a global pandemic, the risk of in-person treatment far outweighs the benefit (with some exclusions), which has led to a variety of changes in telehealth regulations [85]. The Coronavirus Preparedness and Response Supplemental Appropriations Act 2020 has allowed Medicare patients to receive telehealth services in their homes, waived HIPAA violation penalties for providers who treat patients on platforms that do not meet HIPAA standards (eg, Skype and Facetime), and provided federal waivers to a number of states to allow providers to treat their patients out of state.

These changes, as well as others, have significantly reduced barriers to telemental health service provision, but they have also increased risks to personal privacy. It may indeed be the case that the risks involved are tolerated by both the patient and clinician alike, especially when telemental health enables access to care for people who do not otherwise have the resources and luxury to regularly attend in-person treatment [8]. However, the use of digital technology generates a large amount of data that can reveal a lot of personal information, especially when data from multiple data sources are combined. All digital technology data have the potential to be considered personal health information [86]. In addition, there are unresolved issues surrounding the reidentification, marketability, and invisibility (ie, people being unaware of how their data are being used and tracked) of digital technology data [87,88]. Experts in digital research and privacy regulation have a difficult time understanding the risks of using digital technology, and there are no concrete laws to effectively regulate privacy surrounding digital technology, which is constantly evolving [86]. We expect these challenges to be even greater for practicing clinicians and their patients. In order to facilitate transparency, informed consent resources have become available, such as the Telehealth Consent Teach-back Documentation sheet developed by the Agency for Healthcare Research and Quality [89].

### How Can We Prepare Clinicians for the More Effective Use of V-TMH and Development of More V-TMH–Based Treatment Models?

Since the skills used in face-to-face sessions do not necessarily translate to V-TMH sessions, recent studies on mental health professionals have indicated the need for training to deal with clinical, legal, and technical issues [9,90]. Clinicians may be particularly challenged by emergencies in a V-TMH environment [9]. Driven by the relaxing of regulations, the need for an immediate pandemic response has led to the rapid and successful deployment of V-TMH in large academic centers [1] and clinical training programs [91]. These case studies have identified important recommendations for optimizing the physical arrangement of sessions (eg, eliminating visual and auditory distractions), setting up technology (eg, reducing open programs on one’s screen), and communicating effectively with patients (eg, clarifying expectations, benefits, and backup plans in case access to V-TMH is lost).

Expanded V-TMH deployment may also require the consideration of new specialties or service offerings, such as those provided by professional clinical technologists (ie, individuals familiar with a wide variety of digital health resources) [92]. Their main functions would be to match and train patients on health technologies that address their clinical needs, as well as train, educate, and support care providers. The development of web-based clinics is another potential vehicle for delivering digital care, as treatment would be constructed around the use of technology with patients while increasing the access to and offerings of clinical services [93].

## Conclusion

V-TMH, as well as telemental health in general, has a great amount of potential for providing opportunities to increase access to mental health care. However, with the COVID-19 pandemic, the use of V-TMH has been forcefully expanded to those who might benefit more from in-person mental health care. Understanding V-TMH users’ perspectives can help shape future services to be rendered and delivered in a safe and effective manner. This paper provides expert opinions from those with expertise in clinical psychology, service design, and research. Given the uncertain future of the COVID-19 pandemic, addressing the important questions presented in this paper will be critical for maximizing the value of V-TMH for patients and providers. V-TMH may have been disseminated through forced use, but it is unlikely to disappear once the current public health crisis has ended. The issues we have described will have strong implications for technology innovation, the adaptation of treatments to new technologies, and training professionals in delivering V-TMH services and other digital health interventions. Regulations and reimbursement policies need to encourage the broader use of V-TMH, which has the potential to expand access to treatment. The infrastructure for using V-TMH services needs to be better developed; people need reliable internet access and technology with the capabilities for V-TMH. This is an issue of social justice and should be a primary concern. High-speed internet, innovative delivery tools, and training programs for professionals who use such tools can equitably address the mental health needs of the currently served, underserved, and unserved.

## Abbreviations

APA: American Psychological Association
CMC: computer-mediated communication
DOORS: Digital Opportunities for Outcomes in Recovery Services
EHR: electronic health record
HIPPA: Health Insurance Portability and Accountability Act
IPV: intimate partner violence
V-TMH: videoconferencing-based telemental health
